# Molecular Features in Lymphatic Metastases Reflect the Metastasis Mechanism of Lymph Nodes With Non-Small-Cell Lung Cancers

**DOI:** 10.3389/fbioe.2022.909388

**Published:** 2022-07-18

**Authors:** Nannan Guo, Yuanyuan Chen, Zhongying Jing, Siyao Liu, Junyan Su, Ruilin Li, Xiaohong Duan, Zhigong Chen, Ping Chen, Rongjiang Yin, Shaojun Li, Jian Tang

**Affiliations:** ^1^ Department of Thoracic Surgery, Fourth Medical Center of PLA General Hospital, Beijing, China; ^2^ Department of Ultrasound, Fourth Medical Center of PLA General Hospital, Beijing, China; ^3^ ChosenMed Technology (Beijing) Co., Ltd., Beijing, China; ^4^ Computer Network Information Center, Chinese Academy of Sciences, Beijing, China; ^5^ Department of Thoracic Surgery, Yantai Affiliated Hospital of Binzhou Medical University, Yantai, China

**Keywords:** NSCLC, lymphatic metastasis, molecular features, *ATR*, *TET2*

## Abstract

Lymphatic metastasis influences clinical treatment and prognosis of patients with non-small-cell lung cancer (NSCLC). There is an urgency to understand the molecular features and mechanisms of lymph node metastasis. We analyzed the molecular features on pairs of the primary tumor and lymphatic metastasis tissue samples from 15 NSCLC patients using targeted next-generation sequencing. The potential metastasis-related genes were screened from our cohort based on cancer cell fraction. After filtering with gene functions, candidate metastasis-related events were validated in the MSK cohort with Fisher’s exact test. The molecular signature and tumor mutational burden were similar in paired samples, and the average mutational concordance was 42.0% ± 28.9%. Its metastatic mechanism is potentially a linear progression based on the metastatic seeding theory. Furthermore, mutated ataxia telangiectasia mutated and Rad3-related (*ATR*) and tet methylcytosine dioxygenase 2 (*TET2*) genes were significantly enriched in lymphatic metastases (*p* ≤ 0.05). Alterations in these two genes could be considered metastasis-related driving events. Mutated *ATR* and *TET2* might play an active role in the metastasis of lymph nodes with NSCLC. More case enrollment and long-term follow-up will further verify the clinical significance of these two genes.

## Introduction

Lung cancer is the leading cause of cancer-related mortality worldwide, and more than 90% of lung cancers were diagnosed at II or higher stage (Zeng et al., 2021). Distant metastases are still the main cause of poor prognosis and cancer-related death. Lymph nodes nearby the primary tumor, a strong predictor of disease recurrence and a vital factor for the TNM stage, are the most often first metastasizing location (Nosotti et al., 2012). Currently, some research indicated technological barriers to pN1 evaluation in the early stage of lymph node infiltration still exist (Chen et al., 2019; Liu et al., 2019). Thus, the treatment decision in p-N0 non-small-cell lung cancer (NSCLC) patients suffers urgent challenges. Given the crucial clinical significance of lymph nodes, increasing numbers of clinical experts appealed that we must search and grasp new and systematic diagnostic methods to accurately evaluate lymph nodes’ metastasis status, such as combining molecular biomarkers and traditional pathological technology. Therefore, the emerging demand for metastatic lymph nodes in NSCLC is to understand its molecular characteristics and to identify the potential metastasis-driving events, which would benefit the clinical diagnosis, prognostic evaluation, and treatment strategy for NSCLC patients.

Some researchers were focusing on the heterogeneity of primary sites and metastatic lymph nodes in given gene alterations or expressions (Park et al., 2009; Li et al., 2017) or the metastasis biomarker analysis using unpaired NSCLC samples (Xi et al., 2006; Liu et al., 2019). As currently known, the paired analysis could clarify whether particular events enriched in metastases or primary sites within an individual and disentangle whether events occurred in timely order (Turajlic and Swanton, 2016). Even more, paired analysis of primary-metastasis sites helps settle whether a single primary or metastatic biopsy is sufficient to make proper treatment decisions. Studies of inter-tumor heterogeneity in matched primary metastases have revealed that most driver gene alterations tend to be clonal and thus exist in every metastatic site (Reiter et al., 2018; Turajlic et al., 2018). While the majority of driver gene alterations may be clonal, metastasis-specific driving genes have also been identified in some solid tumors (Brastianos et al., 2015; Hu et al., 2019), some of which are clinically relevant. Up to now, no studies systematically analyzed both molecular characteristics and metastatic process of lymph nodes by matched NSCLC samples.

Here, we comprehensively investigated the genetic divergence between primary tumor and lymph node metastasis with paired tissue samples of NSCLC patients and innovatively explored the lymph node metastasis-driving genes following the metastatic seeding idea rooted in the tumor clonal evolutionary theory. A total of 15 paired primary-metastasis samples were performed with targeted panel sequencing. Furthermore, smoking status was recorded for all enrolled cases. Of these 15 cases, 12 were smokers including current smokers and former smokers, and three patients were nonsmokers. All tissue samples were obtained before any systemic therapy. Our aims included the following: 1) to determine whether the lymph node metastasis of NSCLC has distinct molecular characteristics in terms of single nucleotide variants (SNVs) from their primary tumors; 2) to identify “metastasis-related driving genes” that enabled metastasis of lymph nodes.

## Materials and Methods

### Sample Collection and Data Sources

Primary and matched lymph node metastatic site tissues were collected from 15 NSCLC patients at the Fourth Medical Center of PLA General Hospital. Any cases included met the following criteria: patients were diagnosed as NSCLC with lymph node metastasis. This project was approved by the institutional review board of our hospital. All enrolled patients signed the informed consent forms before sample collection. To verify “metastasis-related driving genes,” the Lung_MSK_2017 NSCLC cohort was obtained from the Genomic Data Commons (GDC) data portal (http://gdc-portal.nci.nih.gov). Mutation data in “maf” files were downloaded from The Cancer Genome Atlas (TCGA, http://gdc-portal.nci.nih.gov/) database, which included 492 lung squamous cell carcinoma (LUSC) and 567 lung adenocarcinoma (LUAD) samples.

### Sequencing and Variant Analysis

Genomic DNA extraction and library preparation with TruSight™ Oncology 500 (TSO 500) Library Preparation Kit (Illumina, San Diego, CA, United States) were performed following protocols described previously (Li et al., 2021). The library was sequenced on the Illumina NextSeq 550Dx platform with the paired-end run of 150 base pairs. The quality of sequencing data was verified by TSO 500 Docker pipeline. The process of SNVs and Indels mutation calling, TMB measuring, and reads filtering were performed following the description in the previous study (Li et al., 2021). Germline variants could be filtered out using an in-house built database, and all parameters were set according to the previous workflow (He et al., 2021).

### Mutational Signature Analysis

The COSMIC (the Catalogue of Somatic Mutations in Cancer) mutational signatures analysis was performed to discover the mutational signature difference between primary lesions and lymph node metastases (Jiang et al., 2021). The R package “MutationalPatterns” (Blokzijl et al., 2018) was used to characterize and visualize the analysis results of the *de novo* mutational signatures inference from nonnegative matrix factorization (NMF) in our cohort. The hierarchical clustering analysis was performed to classify lung cancer samples into two subclusters based on signature contribution proportions. We then mapped these two *de novo* signatures to the known COSMIC signatures by the Pearson correlation analysis.

### Identification of Metastatic Drivers

First, the cancer cell fraction (CCF) of somatic mutations in paired samples was calculated using the CCF function of the “cDriver package” (Zapata et al., 2017). Second, mutations were classified into two groups, clonal mutations with CCF >0.6 and otherwise subclonal mutations, which were consistent with previous studies (Hu et al., 2019; Tang et al., 2021). Candidate metastasis-related genes, which first occurred in lymph nodes or clonal in metastasis and subclonal in primary sites, were listed. Third, candidate metastasis-related genes, which are conducive to tumor formation, development, and metastasis, are screened out based on gene function. Lastly, on the aforementioned chosen genes were performed the enrichment analysis, which compared the mutational prevalence in primaries and metastases, with an unpaired cohort, Lung_MSK_2017. The distribution of alterations in *ATR* and *TET2* was analyzed by Maftools.

### Statistical Analysis

Then R package “maftools package” (Mayakonda et al., 2018) was applied to perform the mutation analysis and provide a visualized process of variant analysis results. All statistical analyses were performed by R version 3.6.3. We applied the Pearson correlation analysis to calculate the mutational concordance of P-LN (P: primary, LN: lymph node) samples. For categorical variables, a one-tailed Fisher’s exact test was used. All reported *p* values were two-tailed, and *p* values <0.05 were considered statistically significant.

## Results

### Overview of Our Cohorts

To clarify the molecular features of lymphatic metastasis, we recruited 15 advanced lung cancer patients in clinical stage II ∼ IV (Table 1). Of the enrolled patients, 13 were male and more than 50% were older than 66. A total of 15 patients had matched P-LN (P: primary, LN: lymph node) pairs. We also recorded the smoking status of patients. Smoking patients account for 80.0% (12/15), including current and previous smoking patients. Considering selection pressure, each tissue was collected before systematic treatments. We then conducted targeted sequencing for these 30 specimens obtained from 15 NSCLC patients. After mapping and filtering steps, a total of 294 somatic single nucleotide variants (SNVs) were applied to the following analysis in our cohort. The basic information of the enrolled cases, including patients’ characteristics, tumor mutational burden (TMB), and mutation numbers are listed in Supplemental Table S1. In addition, the Lung_MSK_2017 cohort was applied to verify “metastasis-related driving genes” which were screened from the paired cohort. Notably, SNVs alterations with CCF greater than 0.6 were classified as clonal and subclonal otherwise (Tang et al., 2021).

**TABLE 1:** Clinical characteristics of NSCLC patients.

Patients’ characteristics (*N* = 15)	
	n (%)
Age, median (range)	64 (47–73)
Sex	
Male	13 (86.7)
Female	2 (13.3)
Clinical stage	
II	4 (26.7)
III	10 (66.7)
IV	1 (6.7)
Smoking condition	
Smoking	9 (60)
Off-smoking	3 (20)
Never-smoking	3 (20)

### The Mutational Pattern of Paired P-LN

**FIGURE 1:**
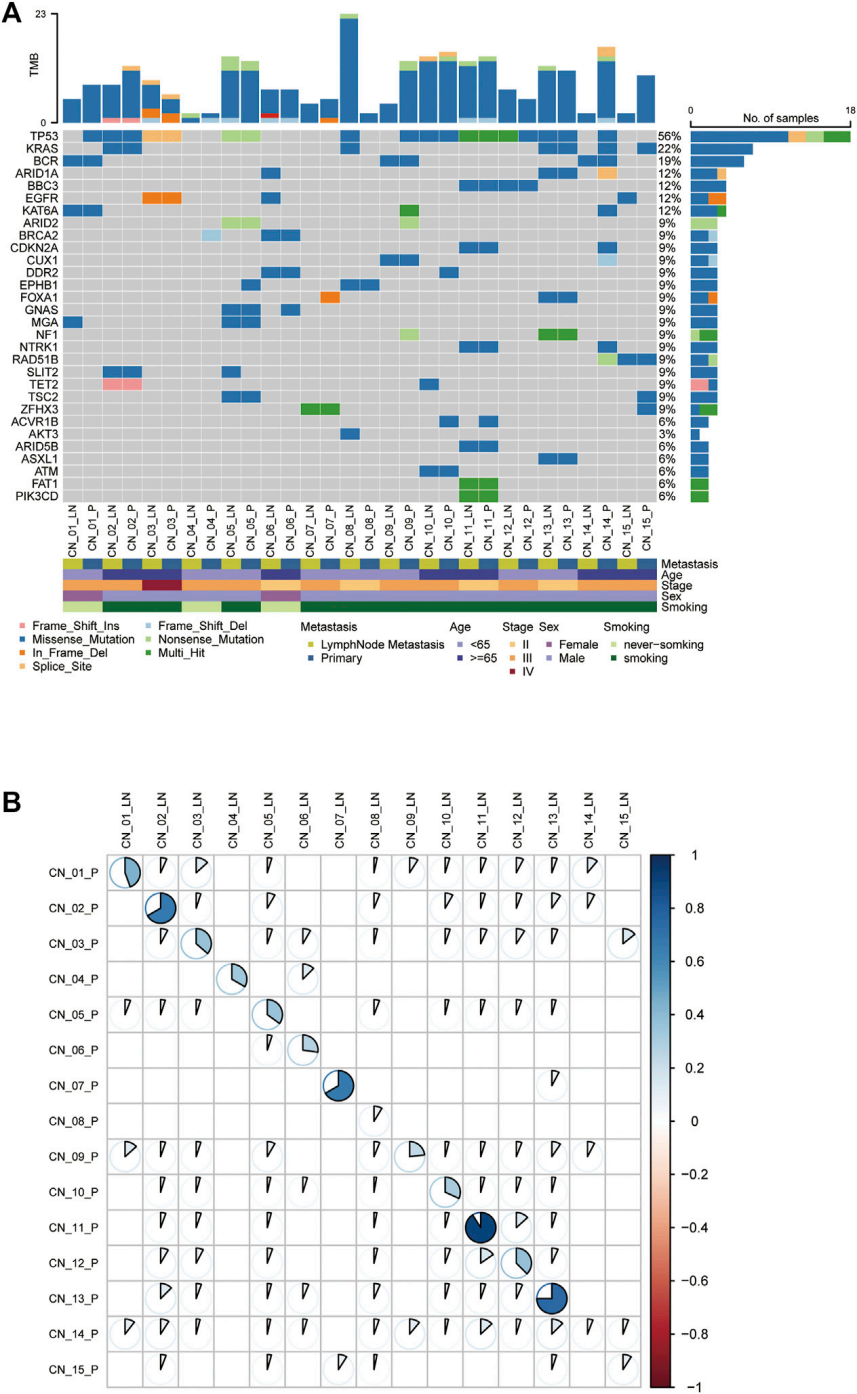
The genetic landscape of metastatic NSCLC patients. **(A)** Comparison of the mutational landscape between primary lesions and their paired lymphatic metastases. The top panel represents the number of somatic mutations in each sample. The middle panel represents the matrix of mutations in a selection of frequently mutated genes. Columns represent samples. The patients’ characteristics are presented in the following. **(B)** The Pearson correlation analysis of mutations in paired P-LN. P: primary lesions; LN: lymph nodes metastases.

Somatic SNVs were identified in each patient varying from 2 to 20 (median, 11) in primary tumors and 2 to 23 (median, 8) in metastases (Supplemental Table S1). The most frequently mutated genes are tumor protein p53 (*TP53*, 56%), Kirsten rat sarcoma viral oncogene homolog (*KRAS*, 22%), and epidermal growth factor receptor (*EGFR*, 12%) mutations were among the top 10 somatic alterations (Figure 1A). Notably, the exhibited mutated genes in the molecular landscape were highly consistent between paired primary and metastatic samples (Figure 1A). With comprehensive consideration of the patients’ smoking status recorded in our cohort, we found alteration counts in one nonsmoker (CN_04) were the least. Regarding nonsmoking lung cancer patients, the counts of molecular alterations in primary and metastatic lymph nodes varied from 2 to 8 (median number, 7) and 2 to 7 (median number, 5), respectively (Supplemental Table S1). Mutation counts in smokers’ primary lesions range from 2 to 20 (median number, 12), while in lymph node metastases range from 2 to 23 (median number, 9) (Supplemental Table S1). Despite the lack of sufficient statistical significance, nonsmokers present fewer molecular alterations than smokers regardless of primaries or metastatic lymph nodes (Supplemental Table S1), which was consistent with the opinion that never-smoking cancers may show a lower mutational burden (Tang et al., 2021).

### Concordance of Paired P-LN

To explore inter-tumor heterogeneity, molecular alterations identified were classified into shared (mutations in both primaries and metastases) and private (mutations in unique primaries or metastases) groups (Jiang et al., 2021). Shared mutations were used to calculate concordance between primary and metastases. The average concordance frequency in our cohort was 42.0% ± 28.9%, ranging from 5.9% to 95.0% (Figure 1B). Of note, three patients exhibit a lower proportion of P-LN shared alterations less than 10%, including CN_08 (8.7%), CN_14 (5.9%), and CN_15 (9.1%), whereas the others were higher than 20% (Figure 1B). We found all these three patients with lower concordance are smokers. This result might suggest that smoking lung cancer patients are apt to have higher heterogeneity between primary tumor and lymph node metastases. Thus, our results revealed that lung cancer presents have higher heterogeneity between primary tumor and metastatic lymph nodes if the cutoff of shared mutations is defined simply as 50%, while individual differences cannot be ignored.

### TMB Analysis of Paired P-LN

**FIGURE 2:**
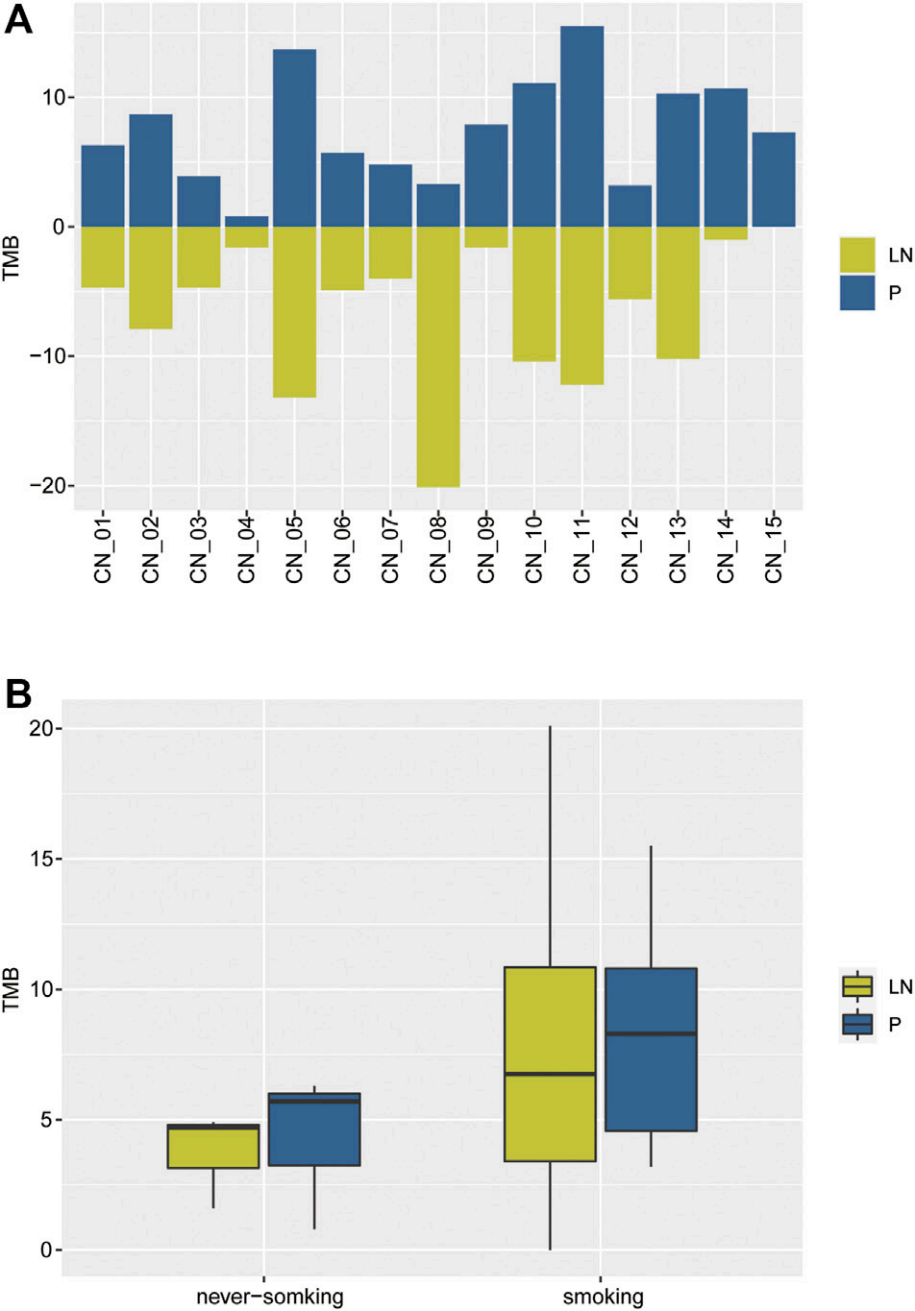
The distribution of TMB of the NSCLC paired samples. **(A)** The comparison of TMB between primary and lymph nodes metastatic samples. **(B)** The comparison of TMB in primaries and lymphatic metastases between smoking and never-smoking NSCLC patients.

As a developing biomarker for NSCLC, TMB presents organ-specific properties (Stein et al., 2019). TMB was then calculated, ranging from 0.8 to 15.5 with a median value of 7.3 in the primary tumor cohort, and 0 to 20.1 with a median value of 4.9 in metastatic lymph nodes (Figure 2A). Never-smoking NSCLC patients acted out lower TMB than smokers but without statistical significance (due to smaller sample number), irrespective of primary or metastatic lymph node (Figure 2B). Moreover, 4/13 of smoking patients showed significant divergence more than two times in TMB of paired P-LN samples, including CN_8 (7.9, 1.2), CN_9 (7.9, 1.6), CN_14 (10.7, 1), and CN_15 (7.3, 0) (Figures 2A,B).

Our results suggested the TMB of lymphatic metastases (median, 4.9) was lower than primary tumors (median, 7.3) but without statistical significance due to the limited samples. Moreover, a few smoking NSCLC patients exhibited inter-tumor heterogeneity.

### Mutational Signature Analysis of Paired P-LN

**FIGURE 3:**
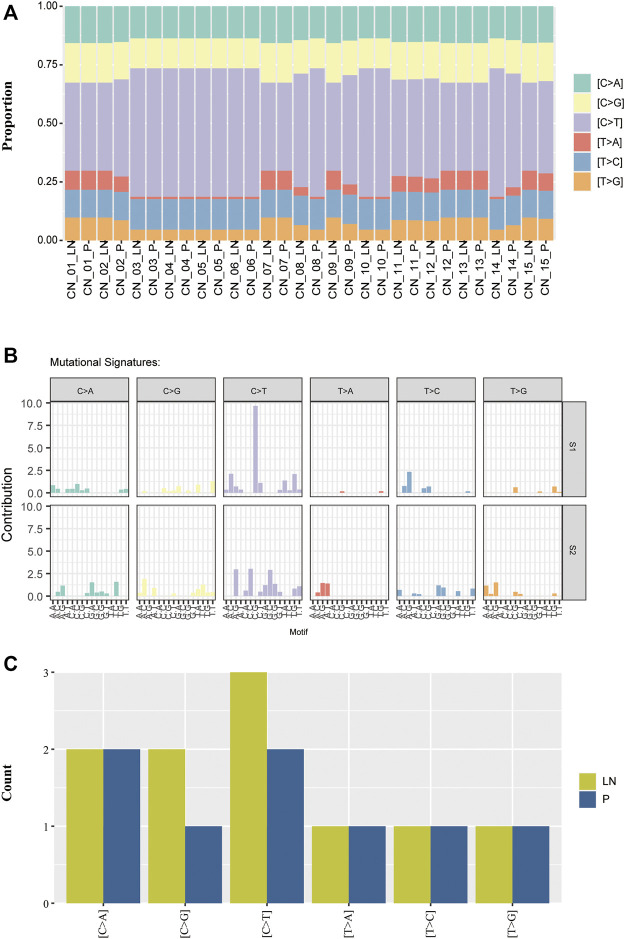
The distribution of mutational signatures of the NSCLC paired samples. **(A)** Comparison of mutational signatures between primary and lymph nodes metastatic samples. **(B)** Two distant mutational signatures were identified by the NMF analysis of the matrix of mutational proportion across tumors from primary and metastatic lesions. **(C)** Comparison of mutational signatures of the private alterations between primary and lymph nodes metastatic samples.

As different mutational processes generate unique mutational signatures, the mutational signatures were analyzed with paired P-LN samples. Each case was dominated by C > T transversion without an obvious difference (Figure 3A). The majority of paired P-LN (12/15) shared the same mutational signature, whereas three paired samples (CN_8_P/LN, CN_9_P/LN, and CN_14_P/LN) present different signature patterns (Figure 3A). By the usage of the Mutational Patterns R package (Blokzijl et al., 2018), our cohort was classified into two groups (Figure 3B). Compared to the S2 signature, the S1 signature present decreased proportion of T > A which is accompanied by an increase in the C > T pattern (Figures 3A,B). Furthermore comparisons between these two signatures with COSMIC-defined signatures (https://cancer.sanger.ac.uk/cosmic/signatures_v2) were made, which was useful to uncover the pathological and clinical relevance of these two signatures. These seven paired P-LN samples and CN_9-LN were clustered to the S1 signature matched COSMIC signature 1, which is found in all cancer types and correlates with the age of cancer diagnosis (Figures 3A,B). The other samples were dominated by the S2 signature closing to COSMIC signature 23, whose function was still unclear (Figures 3A,B).

We further analyzed mutational signatures of private alterations in primary and lymph node metastases. The results suggested that the number of the six types of transversions in primary private mutations was equal to that in LN private mutations (Figure 3C). These results indicated that matched P-LN lesions of lung cancer patients have a similar mutational process to primary tumors.

### Metastatic Driving Gene Analysis

Taking advantage of the matched P-LN samples in our cohort, we attempt to search for metastasis-related events that were first seemed in metastatic lymph nodes or changed from subclone (in the primary tumor) to clone (in metastasis) (Tang et al., 2021). Then 55 SNVs with clarifying clinical and pathological or ambiguous functions were selected as metastasis-related events (Supplemental Table S2). Regarding the functions of genes in NCBI (https://www.ncbi.nlm.nih.gov/gene), we performed a secondary screen to limit the SNVs to metastasis-driving genes, which can promote or drive solid tumorigenesis. Thus, 17 potential metastasis-related driving genes were produced and their mutation frequencies in our cohort and the Lung_MSK_2017 cohort are shown in Supplementary Figure S1. Furthermore, we compared the mutation frequencies of the 17 potential metastasis-related driving genes in lung adenocarcinoma and lung squamous cell carcinoma downloaded from The Cancer Genome Atlas (TCGA) database, and no significant difference was found as shown in Supplementary Figure S2.

**FIGURE 4:**
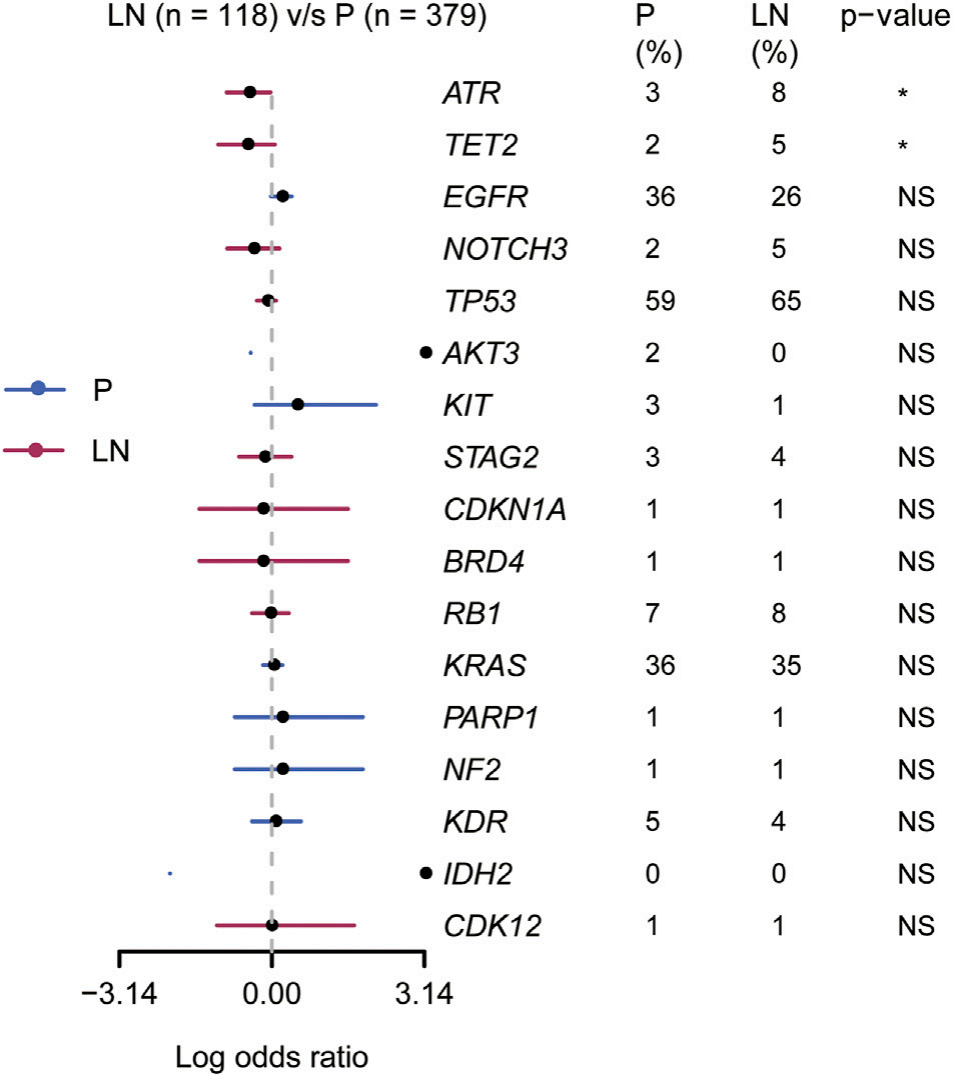
Association of 17 candidate metastasis-related driving genes with primary and lymphatic lesions in the Lung_MSK_2017 cohort.

Based on clonal evolutionary theory, metastasis-related driving genes would be enriched in metastases. Thus, we performed the gene enrichment analysis in the Lung_MSK_2017 cohort with unpaired samples. As shown in Figure 4, *ATR* and *TET2* (2/17 of candidate metastasis-related genetic features) were found significantly enriched in metastatic lymph nodes compared to primary tumors (*p* < 0.05). We also tried to explore whether lymphatic metastasis has a different mutational domain or cluster from the paired primary tumor, but the results were disappointing (Supplementary Figures S3A–D). These results suggested that *ATR* and *TET2* were lymphatic metastasis-related driving genes for NSCLC patients.

## Discussion

Recurrence rates of nodal negative NSCLC are various from 25% to 50% and survival improved in these patients who received adjuvant chemotherapy (Johnson and Rabin, 2005), which hints the routine staging of lymph nodes is imprecise. Nonetheless, metastatic lymph nodes play a crucial role in diagnosis, treatment decisions, and prognosis for NSCLC patients. It appears emergent to understand the molecular features and metastasis mechanism in metastatic lymph nodes. Here, we analyzed the genomic characteristics and metastasis mechanism of metastatic lymph nodes with paired samples.

Our results demonstrated that the metastatic lymph nodes share similarities in mutational signatures and TMB with primary tumors. The average rate of concordance of paired P-LN samples is close to 50%, consistent with the previously published data (Tang et al., 2021). The genetic concordance of the paired primary and lymphatic metastasis would increase if more primary and metastatic regions were collected based on the metastatic evolution theory (Turajlic and Swanton, 2016). Thus, we ventured to guess that the metastatic process of lymph nodes with cancer cells is likely to be a linear progression. At the initial stage of metastasis, the genetic characteristics of lymph nodes are similar to primary lesions, and thus develop genetic diversity over a long evolutionary time. It should be noted that the concordance of lymphatic metastasis was lower than other common metastases in lung cancer, such as metastatic bone and brain (Tang et al., 2021). Thus, we infer local lymph node metastases may have similar growth environments with lung cancer primaries based on their spatial distance following the seed and soil hypothesis (Paget, 1989) and clonal evolutionary theory (Turajlic and Swanton, 2016).

Mutational signatures were applied to clarify different mutational processes which cause different mutation types in most cancer classes (Alexandrov et al., 2013). Somatic mutations of lymphatic metastases were therefore analyzed to define mutational signatures which include six categories of base substitutions (T > A, T > C, T > G, C > A, C > G, and C > T), and compared with primary lesions. Similar mutational signatures of P-LN were observed in 80% (12/15) of NSCLC patients, which further proved our conclusion that lymph node metastasis was more consistent with the linear metastasis mechanism. So far, no studies systematically analyzed both molecular characteristics and metastatic process of lymph nodes by matched NSCLC samples. With the continuous enrollment of samples, we will further study the metastasis model of other distant metastases, such as liver metastasis and brain metastasis, and systematically analyze how NSCLC transfer from local to distant sites.

Our primary aim is to clarify metastasis-related biomarkers to elongate the understanding of the lymphatic metastasis mechanism. Up to date, the regular analyzing methods for metastatic events include analyzing unpaired samples in a large cohort (Shih et al., 2020; Nguyen et al., 2022) and evolutionary analysis in a smaller cohort with paired samples (Jiang et al., 2021). In our study, we screened metastasis-related events with 15 paired samples and then verified them in the Lung_MSK_2017 NSCLC cohort. First, 17 genes were identified as clonal or private alterations in lymphatic metastasis; second, the alteration frequencies of *TET2* (2% in P, 5% in LN) and *ATR* (3% in P, 8% in LN) were significantly higher in lymphatic metastasis than primary lesions, which considered as metastasis-related events. To exclude the influence of different pathological subtypes of NSCLC, we analyzed the frequencies of 17 metastasis-related genes in LUAD and LUSC from the TCGA database. We also remarkably noticed the limitation in our study that there is no CNVs analysis involved in our research. However, unpaired but large samples cohort analysis suggested that distant molecular mutational features may drive metastasis of specific organs (Nguyen et al., 2022).

Of these two potential metastasis-related biomarkers, *ATR*, a protein responding to DNA damage, is mutated at a high frequency (8%) in lymphatic metastasis than in primary tumors (3%) with lung cancers, but there is no difference in *ATR* alteration sites between primary and lymphatic lesions. The ATR protein, first identified as a mammalian ortholog of Mec1 in 1996 (Cimprich et al., 1996), together with ATM (ataxia telangiectasia mutated protein) and DNA-PKcs (DNA dependent protein kinase catalytic subunit) form the core of the DNA damage response (DDR) (Jackson and Bartek, 2009). These proteins have been conventionally regarded as tumor suppressors whose functional loss can give rise to cancer-promoting mutations and generate neoantigens. Importantly, *ATR* was regarded as the primary sensor of oncogenes that triggered replication stress and DNA damage. Previous studies revealed that *ATR* was likely to be a prognostic biomarker for some solid tumors (Feng et al., 2020), but the result was contradictory found by Zighelboim et al. (2009). Kashyap et al. (2020) found that *ATR* presented with a low transcriptional level in 76% of uveal melanoma patients who were significant with metastasis (*p* = 0.046). In one case report, mutated *ATR* and *BRCA2* were thought to contribute to extensive metastasis with clear renal cell carcinoma (Yang et al., 2021). Collectively, *ATR* could be a metastasis-related biomarker and a prognostic biomarker in NSCLC patients but needs to be further proved. *TET2* is another potential lymphatic metastasis-related biomarker identified in this study with higher mutation frequency in metastatic lymph nodes than in primary tumors and no obvious difference in the mutational domain. TET2 is a dioxygenase that belongs to the TET family proteins (TET1, TET2, and TET3) catalyzing 5-methylcytosine (5 mC) into 5-hydroxymethylcytosine (5 hmC) in DNA (Pastor et al., 2013). No evidence indicates that mutated *TET2* is associated with lymphatic metastasis, although it promotes liver and brain metastasis with lung cancer (Li et al., 2020; Dono et al., 2021; Nguyen et al., 2021). Another study in prostate carcinoma suggested that *TET2* was associated with aggressive disease with a mutation frequency of 24.4% in aggressive prostate cancer patients and 9.6% in disease-free control (Koboldt et al., 2016). In this study, *TET2* was shown to have the potential of promoting NSCLC lymph nodes metastasis. Our research is still ongoing. In the future, we will further analyze the activity and expression of the ATR/TET2 protein in NSCLC patients with or without mutated *ATR* or *TET2* and analyze the impact of mutated *ATR*/*TET2* on the prognosis and survival of patients. It is urgent to clarify whether these two genes could guide the dissection of lymph nodes, especially for those whose lymph nodes are identified as pathological naive.

Several restraints should be acknowledged. First, we collected the primary or metastatic tissue from one region instead of multi-regions. To abate the interference of intratumor heterogeneity, it is ideal to validate our results with multiregional samples (Jamal-Hanjani et al., 2017). Second, we used TSO 500 targeted panel to characterize the genetic alterations of matched P-LN samples. Targeted sequencing might miss some important genes and structure variants as whole genome sequencing could give unbiased results. Third, when we analyzed the metastases enriched genetic alterations, our cohort was too small while this disadvantage was overcome by the large sample size of the MSK cohort. Thus, it is better to confirm our results in the large paired cohort. Fourth, what cannot be ignored is the patient’s characteristics, gender, age, pTNM stage, and smoking history. These differences in patient characteristics may produce results bias, which means we excessively highlight the genetic changes despite the cohort difference.

In summary, our study suggested that the metastatic process of lymph nodes is the result of the linear progression, and *ATR* and *TET2* are likely to play a crucial role in NSCLC lymph nodes metastasis. This finding would deliver new insights into the process of lymph node metastasis and provide suggestive information for developing novel approaches to monitor prognosis, formulate treatment strategy, and further refine pathological staging of lymph nodes.

## Data Availability

The paired sample data of 15 NSCLC patients for this study are included in the article/Supplementary Material. The MSK dataset was collected from the Genomic Data Commons (GDC) data portal (http://gdc-portal.nci.nih.gov).
