# Supplementary material for: Molecular Features in Lymphatic Metastases Reflect the Metastasis Mechanism of Lymph Nodes With Non-Small-Cell Lung Cancers
Source: Front Bioeng Biotechnol. 2022 Jul 18;10:909388. doi: 10.3389/fbioe.2022.909388 (PMC9341247; doi:10.3389/fbioe.2022.909388)
Supplement: Supplementary file 2 [file DataSheet1.docx]

**
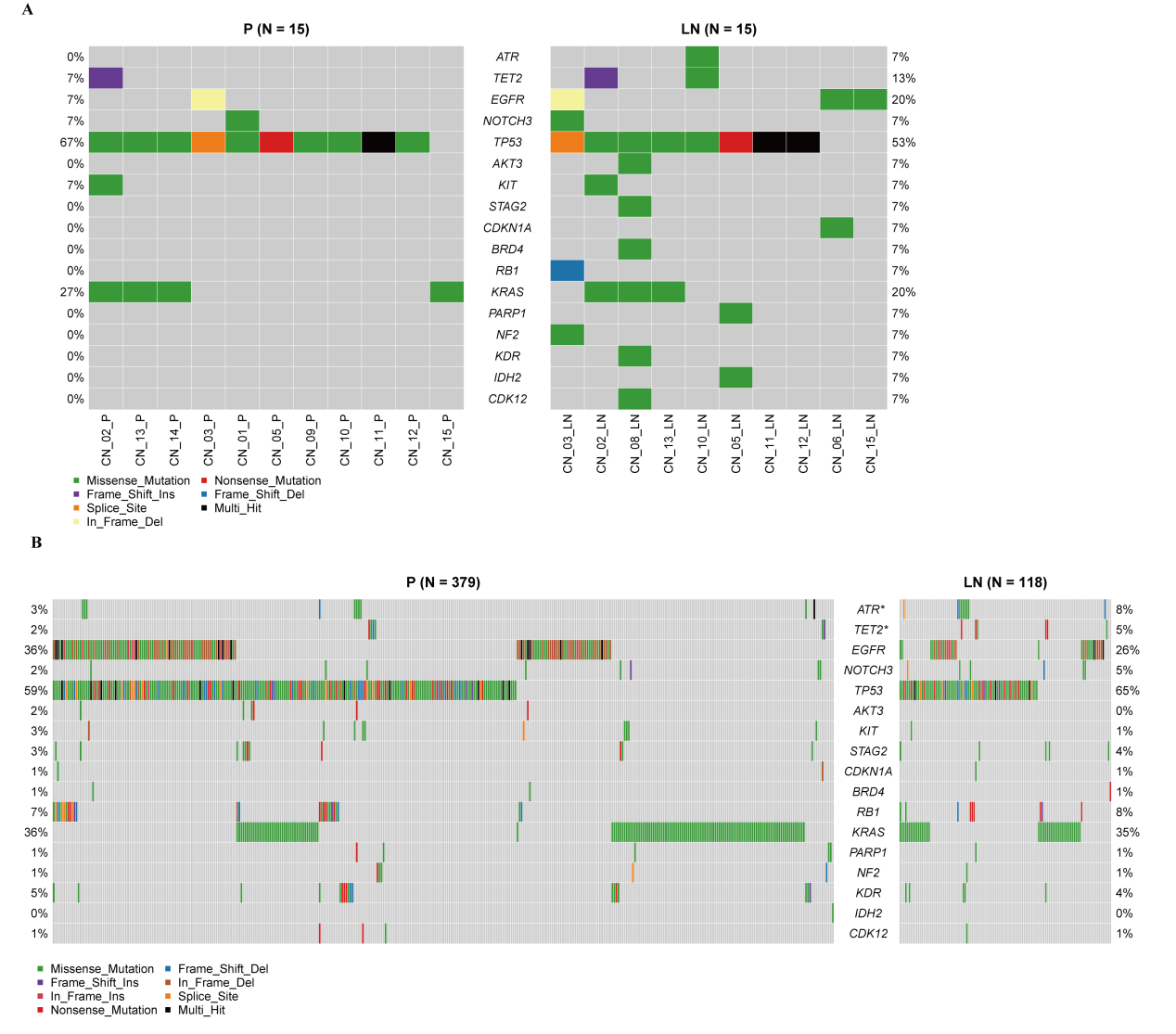
**

**FIGURE S1|** (A) Mutational landscape of 17 candidate metastasis-related driving genes in 15 paired samples. (B) Mutational landscape of 17 candidate metastasis-related driving genes in Lung_MSK_2017 cohort with unpaired samples. The left mutational map presented the distribution of mutational alterations in primary samples. The right mutational map presented the distribution of mutational alterations in lymph node metastatic samples. P, primary lesions; LN, lymph nodes metastases.


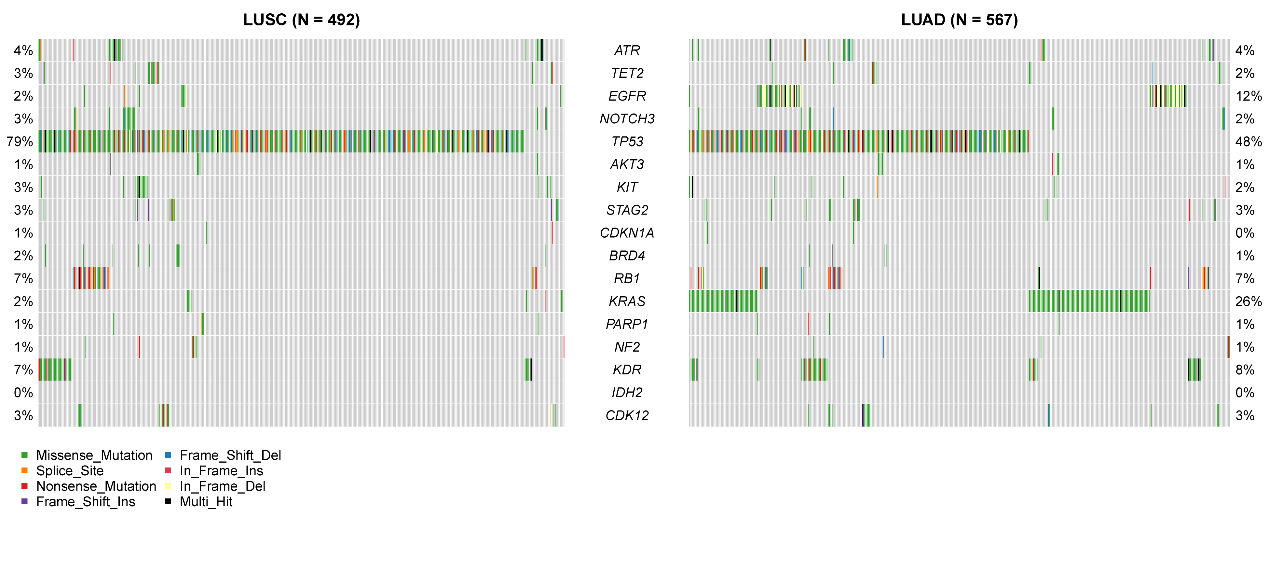


**FIGURE S2|** The mutation frequency of 17 metastasis driving genes in lung adenocarcinoma and lung squamous cell carcinoma from The Cancer Genome Atlas (TCGA) database.


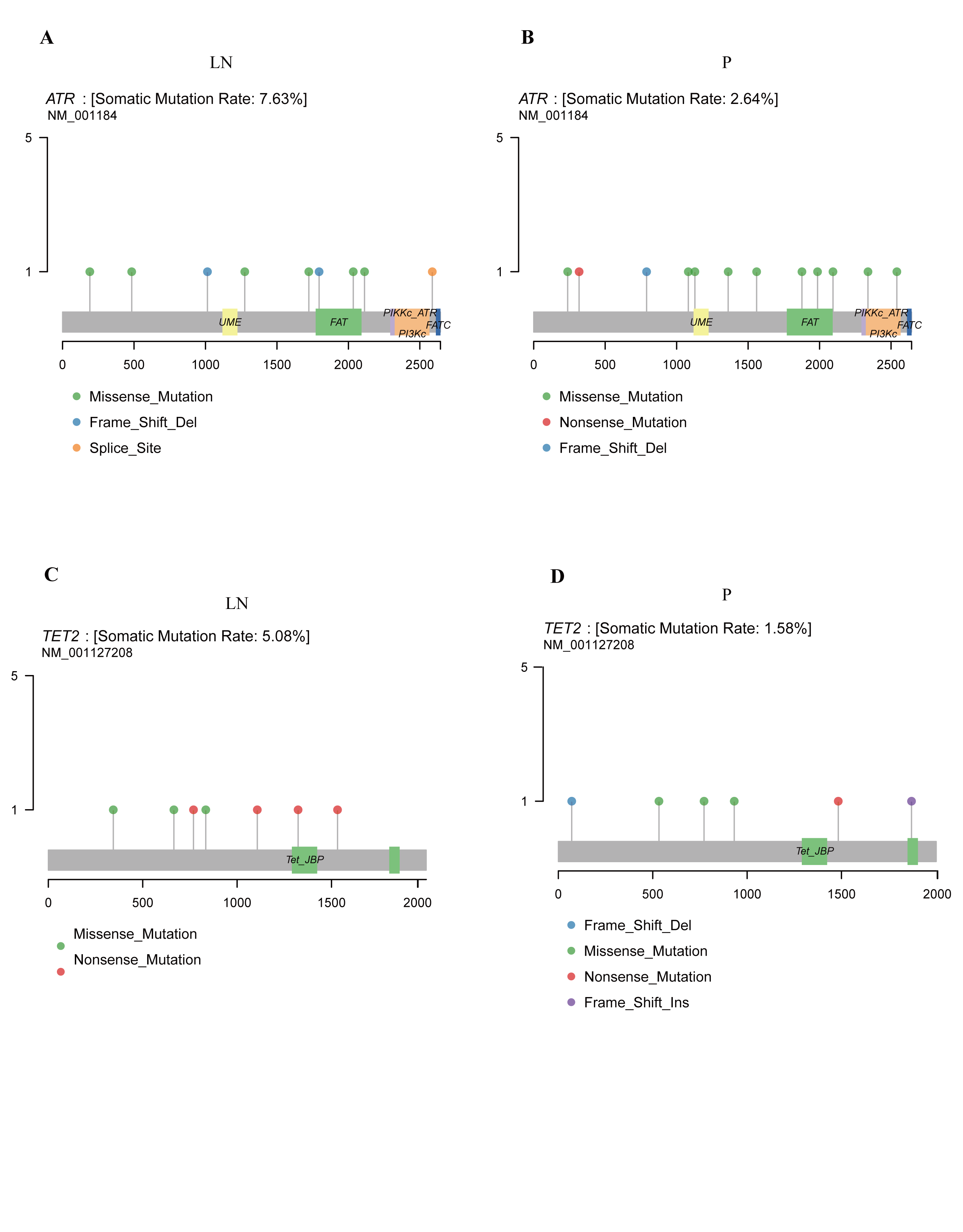


**FIGURE S3|** The distribution of mutational alterations in *ATR* and *TET2*. Mutation sites of *ATR* in metastatic lymph nodes **(A)** and primary samples **(B)** within the Lung_MSK_2017 cohort. Mutation sites of *TET2* in metastatic lymph nodes **(C)** and primary samples **(D)** within the Lung_MSK_2017 cohort. Somatic mutation rates are shown in the title.
